# From description to implementation: key takeaways from the 3rd African Microbiome Symposium

**DOI:** 10.1128/msphere.00683-25

**Published:** 2025-11-19

**Authors:** Charissa C. Marsh, Kristien Nel Van Zyl, Olubukola Oluranti Babalola, Reinhard Böhmer, Don A. Cowan, Kgabo L. M. Moganedi, Itumeleng Moroenyane, Jerolen Naidoo, Abigail Nieves Delgado, Joram M. Posma, Leopoldo N. Segal, Mathabatha E. Setati

**Affiliations:** 1 African Microbiome Institute, Division of Molecular Biology and Human Genetics, Department of Biomedical Sciences, Faculty of Medicine and Health Sciences, Stellenbosch University, Cape Town, South Africa; 2 DSI-NRF Centre of Excellence for Biomedical Tuberculosis Research, South African Medical Research Council, Division of Molecular Biology and Human Genetics, Faculty of Medicine and Health Sciences, Stellenbosch University 26697, Cape Town, South Africa; 3 Food Security and Safety Focus Area, Faculty of Natural and Agricultural Sciences, North-West University 274472 , Mmabatho, South Africa; 4 Department of Life Sciences, Imperial College London, Silwood Park Campus 4615, Ascot, Berkshire, United Kingdom; 5 PathCare laboratories, Cape Town, South Africa; 6 Centre for Microbial Ecology and Genomics, Department of Biochemistry, Genetics and Microbiology, University of Pretoria 56410, Pretoria, South Africa; 7 Department of Biochemistry, Microbiology and Biotechnology, University of Limpopo 37714, Sovenga, South Africa; 8 The Plant Holobiont Lab, Department of Botany and Zoology, Faculty of Science, Stellenbosch University 26697, Stellenbosch, South Africa; 9 Council for Scientific and Industrial Research, Future Production Chemicals Cluster 56417, Pretoria, South Africa; 10 Department of Biochemistry, Genetics and Microbiology, University of Pretoria 56410, Pretoria, South Africa; 11 Department of Human Biology, Faculty of Health Sciences, University of Cape Town 37716, Cape Town, South Africa; 12 Freudenthal Institute, Utrecht University 8125, Utrecht, the Netherlands; 13 Section of Bioinformatics, Division of Systems Medicine, Department of Metabolism, Digestion and Reproduction, Hammersmith Hospital Campus, Imperial College London 4615, London, United Kingdom; 14 Division of Pulmonary, Critical Care, and Sleep Medicine, NYU Grossman School of Medicine 12296 , New York, New York, USA; 15 South African Grape and Wine Research Institute, Stellenbosch University 26697, Stellenbosch, South Africa; Shenzhen Institute of Synthetic Biology, Chinese Academy of Sciences, Shenzhen, China

**Keywords:** african microbiomes, metagenomics, capacity building, translational research

## Abstract

The 3rd African Microbiome Symposium was held in Cape Town, South Africa, from 20 to 22 November 2024. The symposium featured a diverse range of local and international microbiome research and provided a platform for 79 researchers, students, and industry members to engage in discussions on the microbiome within an African context and focusing on translational research. This meeting review shares highlights, findings, and recommendations derived from the event. Insights from two panel discussions revealed key barriers to microbiome research in Africa, including limited funding, infrastructure gaps, and a shortage of trained local scientists. Recommendations centered on increased investment, institutional training, adherence to ethical guidelines, and the fostering of equitable global partnerships.

## INTRODUCTION

The African Microbiome Institute (AMI), based in the Division of Molecular Biology and Human Genetics, Faculty of Medicine and Health Sciences, Stellenbosch University (SU), is a cross-faculty initiative with a strong focus on building capacity, collaboration, and networking among microbiome researchers in Africa and abroad. The AMI hosted its third African Microbiome Symposium from 20 to 22 November 2024 at SU’s Tygerberg campus, in the Biomedical Research Institute building. The symposium, which comprised delegates from South Africa and other countries, featured research on human, environmental, and animal microbiomes, under the theme “Beyond description: translation, application, and implementation of microbiome research*.*”

The meeting was preceded by a workshop entitled “Exploring cutting-edge tools and technologies for microbiome research” sponsored by Separations Scientific SA (Pty) Ltd. The workshop was attended by 41 postgraduate students, postdoctoral fellows, and early-career researchers, while the symposium was attended by 79 delegates (35% researchers, 18% industry members, 20% postdoctoral fellows, and 27% students).

**Fig 1:**
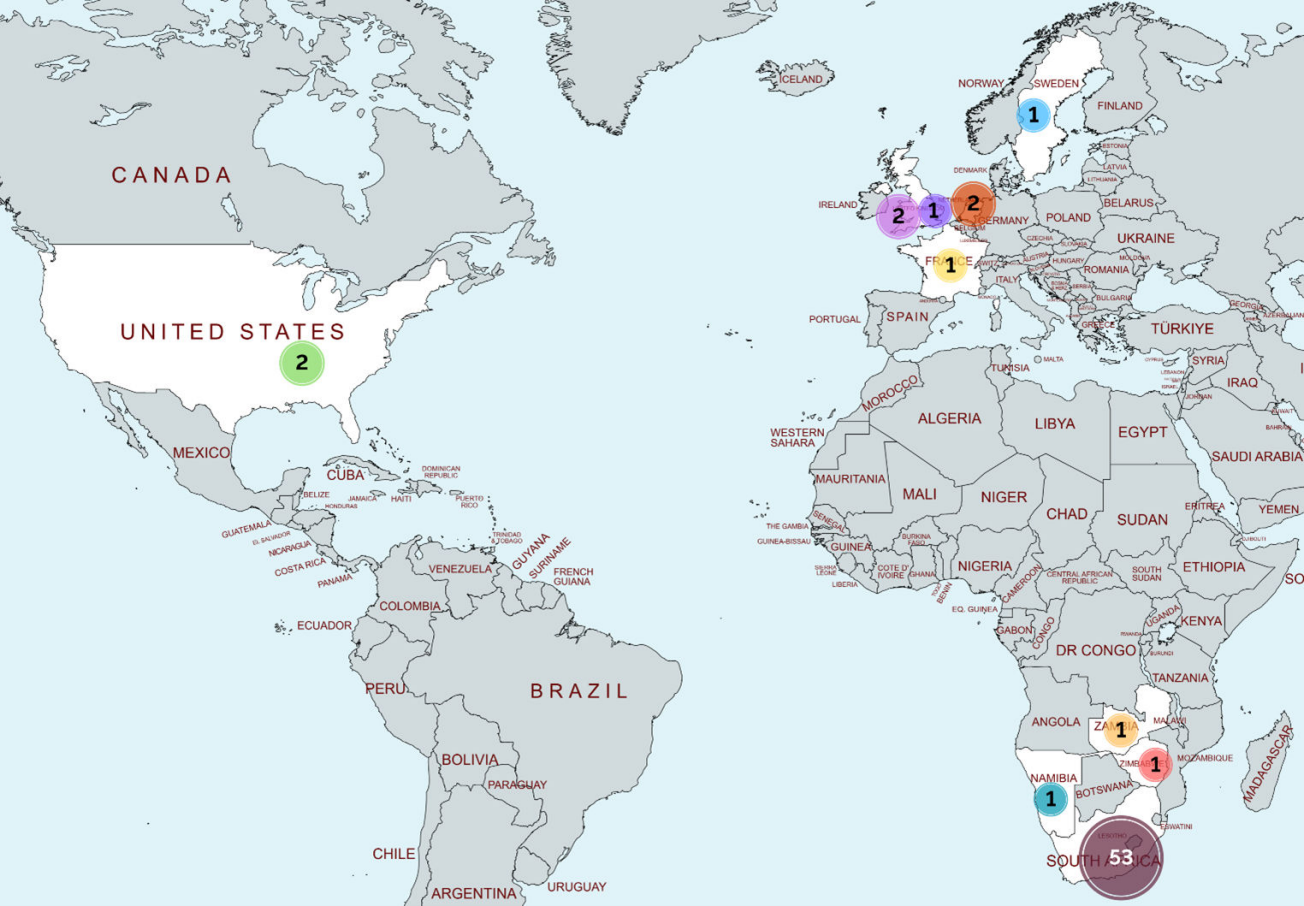
Geographic representation of the 65 academic attendees, including researchers, postdoctoral fellows, and students.

Academics represented 10 countries (Fig. 1), from seven South African institutions (SU, University of Cape Town, North-West University, University of Pretoria, University of Limpopo, University of the Witwatersrand, and University of Zululand), eight international institutions (New York University, University of California San Diego, Utrecht University, Imperial College London, Aberystwyth University, University of Namibia, University of Zimbabwe, and the Karolinska Institute), and three research organizations (the Council for Scientific and Industrial Research [CSIR] in South Africa, the National Research Institute for Agriculture, Food and Environment [WMP-INRAE] from France, and the Tropical Gastroenterology & Nutrition group [TROPGAN] from Zambia).

Following the welcoming address by the SU rector and vice-chancellor, Dr. Wim de Villiers, who emphasized microbiome research as one of the key priority areas at SU and the need for a strong push toward more translational research, a keynote address titled “Unraveling host-microbe interactions in the lower airways through multi-omics” was given by Dr. Leopoldo Segal (New York University, USA). His presentation emphasized the value of study designs that strategically maximize the use of each sample and the integration of methods that monitor microbial transcripts, genes, and metabolites, thereby enabling holistic and reliable functional insights into the lung microbiome that extend beyond basic taxonomy. This was followed by six themed sessions across two days, comprising 18 oral presentations, nine flash poster presentations, and two panel discussions to summarize key takeaways from the day and propose future recommendations. In addition, submitted e-posters were displayed throughout the event and a networking function was held after day 1. The symposium was concluded by Dr. Thulani Makhalanyane (SU) following the presentation of awards for student oral and poster presentations.

## THEMED SYMPOSIUM SESSIONS

The full program, containing the list of talks, posters, and presenters, is detailed in the supplemental material (Tables S1 and S2). Below, we briefly describe the key notes and conclusions from oral presentations in each themed session.

### Insights into microbiomes and human health

The first session highlighted innovative advances in human microbiome research. For example, the CSIR’s Microbiome Mapping Initiative (CMMI) aims to systematically map microbial diversity across different populations and environments in South Africa using both long- and short-read sequencing. This initiative would constitute one of the largest African microbiome data sets to date for inclusion in global resources. The potential of long-read metagenomic sequencing to also enhance diagnosis of infectious diseases was demonstrated in cerebrospinal fluid specimens; however, its clinical utility was limited by low genome coverage and thus requires careful validation. In an intervention-based study (the AMAZE trial), amino acid supplementation showed no clear microbiome modulation although findings were limited by possible duodenal sampling contamination and the constraints of 16S-based functional prediction.

### Microbiomes in agrifood systems

The second session showcased the diversity of plant- and animal-associated microbiota, highlighting how beneficial microbes can improve crop health, fitness, and the quality of indigenous ferments. A vision for resilient agrifood systems in the Global South was presented, emphasizing the critical role of rhizosphere microbiomes in nutrient mobilization, plant protection, and stress resilience. It was noted how studying the microbiome and metabolic function during drought stress can inform sustainable plant protection via engineered interactions with microbes and how beneficial microbes may act as biocontrol agents that contribute to soil fertility and plant growth. Integrating indigenous knowledge with microbiome research can drive innovation and product development, as exemplified by the cultural, nutritional, and socio-economic value of marula fruit wine. Finally, early microbial colonization and fermentative capacity development in ostrich chick digestive development and health were highlighted, identifying risk periods for pathogenic species compromising gut health.

### Flash talks

The third session featured a selection of nine poster abstracts from early career researchers and students, presented in flash presentation format. These posters covered a wide variety of microbiome subtopics, from virus discovery to bacterial involvement in human diseases, antibacterial agents, and natural language processing pipelines for research. During this session, the World Microbiome Partnership (WMP) and their roadmap for One Microbiome Health were also introduced to participants.

### Socio-ethical implications of microbiome research

On day 2 of the symposium, critical topics such as ethics and ethnicity in human microbiome research were brought to the fore during the fourth session. Key issues were raised, such as informed consent and respect for autonomy, data sharing and protection of privacy, as well as the importance of disseminating research-related results to research participants, from a clinician’s perspective. Attendees were encouraged to re-imagine the descriptors used in human microbiome studies, highlighting how some descriptors could lead to racialized views or harmful stereotypes.

### Advances in microbiome analysis

Session five explored a variety of pioneering techniques in microbiome analysis. This included BERT-based large language model (LLM) pipelines for literature review and meta-analysis in microbiome research, indicating high precision and speed compared to open-weight LLMs such as GPT. Bacteriophages lacking genes linked to lysogeny, resistance, and virulence were purported as promising biocontrol agents against virulent and drug-resistant strains of *Escherichia coli* O157:H7 in food safety and clinical settings. A promising proof-of-concept study was presented using metagenomics-based pathogen and antimicrobial resistance (AMR) surveillance on a neonatal platform in hospital settings to inform infection prevention guidelines. Finally, it was shown how combining culture dependent and independent techniques can lead to the discovery and valorization of promising fermentative yeasts species, specifically in pomegranates, for improved wine production.

### Microbiomes in terrestrial and aquatic ecosystems

The final session brought together diverse perspectives on microbial ecosystems. Functional microbiomics techniques were highlighted for identifying the drivers of community structures and health in soil systems, with Namid Desert soils as a successful case study. Seagrass sediment and rhizosphere microbiomes were shown to form distinct but overlapping core communities, with potential for monitoring, restoring, and understanding blue carbon preservation. One talk demonstrated how beneficial microorganisms and their traits could be strategically tracked to construct synthetic ecosystems (SynEco). Finally, using a One Health approach, researchers showed that metabolomic signatures of tuberculosis in both humans and animals can reveal potential biomarkers, linking the microbiome with public health and ecosystem health.

## PANEL DISCUSSION 1: FROM MAPPING TO FUNCTION

Key speakers (Drs. Leopoldo Segal, Jerolen Naidoo, Olubukola Oluranti Babalola, Kgabo Moganedi and Itumeleng Moroenyane) from day 1 participated in a panel discussion chaired by AMI co-director, Dr. Mathabatha Evodia Setati. The panel addressed critical questions around the extraction of meaningful functional relevance from microbiome data, criteria for construction of synthetic communities, as well as challenges and perspectives of moving from mapping to functional microbiome studies.

The panel highlighted that, although there is a wealth of global microbiome data available, leveraging the data for predictive modeling remains a substantial challenge. This is primarily due to disparities in data quality, which may lead to the inability to perform robust analyses, and inconsistencies in the collection of metadata, which mean that many data sets lack depth necessary to address specific research questions. Furthermore, microbiome data from Africa remain highly underrepresented in the global setting despite recent efforts to establish dedicated data repositories in the human research (1). The lack of adequate representation of African microbial diversity in global reference databases has practical consequences for microbiome research across the continent. Specifically, it limits taxonomic resolution when using widely adopted analysis pipelines that rely on these databases. This has a cascading effect, obscuring unique regional signatures, thereby reducing the effectiveness of functional prediction, ecological interpretation, and biomarker discovery, ultimately restricting the generation of local insights and translational potential of African research. This underscores the need for more inclusive data collection and analysis to improve the prospect of predictive modeling and translational capacity from microbiome data in the African context.

Increasing access to sequencing has enabled a broader range of researchers to engage in microbiome research, including those not formally trained in microbiology. While the broadening access dramatically improves the scope of available data, it also raises concerns regarding the adherence to best practices and proper identification of data limitations. For example, background contamination has been recognized as a critical factor in microbiome studies for more than a decade (2, 3), yet many current studies lack appropriate control measures or attempts to identify potential contamination sources (4).

In response to the identified challenges, the panel recommended a more thoughtful research design. This included building scope within studies to address immediate research gaps and enable follow-on studies. In human studies, the microbiome is often studied using convenience specimens (i.e., collected as part of the parent study), which may not fully encapsulate the complexity of microbial diversity at the site of interest or shifts that occur over time. Most microbiome studies in Africa remain largely correlative and focus on profiling microbial communities. The global microbiome community agrees that, to realize translatable impact, research efforts must transition toward uncovering causality and developing implementable solutions as it relates to the microbiome, particularly in the context of human health. Therefore, the panel echoed the opinion that sequencing data (e.g., 16S rRNA gene sequencing, which is commonly applied, but has known limitations) should be complemented with other -omic data sets to draw more meaningful and actionable insights. Additionally, the panel suggested that studies incorporate a comprehensive sample collection (with long-term storage in mind) to facilitate the assembly of specialized biobanks for future microbiome research (and collaboration) using multi-omic approaches.

Microbiome research designs can be particularly complex, and standardization efforts can be impeded by disparities in reagent availability, storage methods, sequencing platforms, and bioinformatics pipelines. However, the panel emphasized the benefits of establishing collaborations that work toward consensus-based guidelines for microbiome research. Partnerships, such as those established within the AMI network, provide structured recommendations that outline minimum requirements and guide researchers to optimal study designs, from sample collection to data analysis. This approach has been successfully demonstrated in the field of lung microbiome research (5, 6). This and other guidelines, such as those of the NIH Human Microbiome Project (7) serve as models for other research domains and demonstrate how community-driven efforts enhance reproducibility and reliability in microbiome science.

Finally, the panel stressed the importance of standardizing metadata (i.e., providing defined criteria) in public data repositories, for comparability and integration with other microbiome studies. Reporting negative results was also encouraged, as the panel felt that this not only promotes transparency and upholds integrity through ethical reporting but also helps normalize failure as a natural and valuable part of the scientific research and would support future research by preventing redundant experimentation efforts.

## PANEL DISCUSSION 2: AFRICAN FOOTPRINT IN MICROBIOME RESEARCH

The panel discussion on day 2 comprised four speakers (Drs. Reinhard Böhmer, Abigail Nieves Delgado, Don Cowan, Joram Posma) and was chaired by AMI co-director, Dr. Charissa Marsh. The discussion focused on ethical, logistical, and technical challenges in African microbiome studies.

The panel recognized that ethical regulations for research are already well-defined in most academic institutions, but that its effective implementation remains the responsibility of individual institutions. The panel emphasized that microbiome researchers bear a responsibility in conducting ethical research that ensures local practices and beliefs are respected and that questions are aligned with the interests and needs of the communities involved, whether it relates to health, agricultural development, or the enhancement of livelihoods. Moreover, the panel emphasized the potential for using participatory research methods wherein the community is allowed to participate and partner in the research, is given an understanding of what is going to be done, how it will be done and what the data will mean for them. By doing this, the likelihood of community participation and cooperation in microbiome research would markedly increase. The continued advancement of ethical African-centric microbiome research is dependent on promoting shared ownership and leadership in studies involving specimens and data derived from African settings. This requires establishing equitable research partnerships with African researchers and communities at the outset, clearly delineating study roles, authorship, and benefit-sharing.

The panel identified several logistical and technical barriers specific to the African context that may limit that advancement of microbiome research. The panel discussed compliance with the Nagoya Protocol (a legally binding international agreement, undersigned by 142 countries, under the Convention of Biological Diversity) (8) which requires researchers to obtain informed consent and establish mutually agreed terms with the country of origin prior to accessing genetic materials (including microbiomes). This is crucial for ethical research but can introduce delays and complexities in sharing data from African settings, particularly if local regulations are not well-defined. The panel acknowledged that increased awareness and structured support for navigating the Nagoya protocol would greatly benefit microbiome researchers working in underrepresented regions of Africa.

Despite the increasing availability of sequencing platforms in Africa in recent years, the cost per sample remains disproportionately high compared to the Global North. This technical barrier limits the local throughput potential and encourages the outsourcing of sequencing to international facilities, which may further limit local capacity building and data sovereignty. The panel further emphasized that while many sequencing hubs have been established on the continent, platforms that enable functional (and multi-omics) microbiome research, such as metabolomics, are not readily available. Furthermore, microbiome expertise, particularly in bioinformatics, remains relatively weak (compared to the Global North). The persistent issue of “brain drain,” where skilled African researchers pursue opportunities abroad due to limited local opportunities and funding, further compounds this challenge and reduces local capacity for independent microbiome research. The brain drain extends beyond academia, limiting opportunities for innovation and the translation of research into industry applications (i.e., nutrition interventions, biocontrol agents, indigenous ferments, and bioremediation) that could otherwise have contributed to economic growth and public health. The panel discussed mitigating strategies, such as integrating microbiome training at the undergraduate level, leveraging international partnerships for postgraduate training and advocating for funding bodies to prioritize capacity development initiatives as part of their funding scheme requirements, particularly if the proposed research is carried out in a resource-limited or lower-middle-income country.

African microbiome research should also be promoted through international collaborations. The World Microbiome Partnership (WMP) (9), which was represented at the AMI symposium, held the One Health WMP Summit on 20 June 2025 and emphasized this type of global cooperation. A declaration, “Harnessing the Power of Microbiomes to Advance One Health,” was made during this summit and specifically addressed the need to build sustainable global microbiome partnerships, identify synergistic opportunities to strengthen innovation in the Global South, and promote equitable data sharing, training opportunities, and geographically balanced participation and leadership (10). The summit concluded with the formation of global working groups to answer the objectives set out in the declaration. Two notable African-led multi-center collaborations that advance African microbiome research include the Africa Wits-INDEPTH Partnership for Genomic Research (AWI-Gen; https://h3africa.org/index.php/awi-gen/), which brings together five African partner institutions alongside two international (Harvard University and University of Bristol), and the African Microbiome Project (https://africanmicrobiomeproject.org/), which focuses on generating microbiome data within the continent, while encouraging equitable partnerships from other international collaborators (11).

In closing, the panel considered strategies to overcome generalized barriers of African microbiome research. These included improving and implementing effective scientific communication with research participants and involved communities to foster interest and demonstrate how the research may improve livelihoods and well-being. Furthermore, demystifying microbiome science for the public would ultimately lead to improved public perception of science and encourage greater involvement in research.

## CONCLUSION

The findings from the African Microbiome Symposium highlight the critical need for a multifaceted approach to advance functional and translational microbiome research in Africa. Several critical barriers were identified, including prohibitive financial costs, limited multi-omics infrastructure, and a shortage of local expertise. These challenges may be overcome by enhanced investment, focused training initiatives (including at an institutional level), and equitable global partnerships that ensure appropriate and continued recognition. This, in turn, will help bridge the considerable gap that remains in translating microbiome research into practical benefits for public health and innovation (such as improved infectious disease management, the development of microbiome-based diagnostics and therapeutics, and bio-based products for sustainable agriculture), and economic development in Africa through advances in biotechnology and bioeconomy sectors. Other recommendations include adopting established and consensus-based guidelines for microbiome research across different domains to ensure ethically sound research, implementing well-thought-out study designs, promoting open communication and engagement with involved communities, and prioritizing capacity development. By addressing these challenges, African microbiome researchers will be positioned as leaders in global health and sustainability, contributing valuable knowledge to the scientific community and empowering local communities. The SU African Microbiome Institute remains committed to this end.

## SUPPLEMENTAL MATERIAL

Tables S1 and S2 msphere.00683-25-s0001.pdf Full program and speaker lists for the 3rd African Microbiome Symposium and pre-symposium workshop.

ASM does not own the copyrights to Supplemental Material that may be linked to, or accessed through, an article. The authors have granted ASM a non-exclusive, world-wide license to publish the Supplemental Material files. Please contact the corresponding author directly for reuse.
