# Supplementary material for: From description to implementation: key takeaways from the 3rd African Microbiome Symposium
Source: mSphere. 2025 Nov 19;10(12):e00683-25. doi: 10.1128/msphere.00683-25 (PMC12724225; doi:10.1128/msphere.00683-25)
Supplement: Tables S1 and S2 — Full program and speaker lists for the 3rd African Microbiome Symposium and pre-symposium workshop. [file msphere.00683-25-s0001.pdf]

From description to implementation: key takeaways from the 3rd African Microbiome Symposium

SUPPLEMENTARY MATERIAL

Charissa C. Marsh[a,b*], Kristien Nel Van Zyl[a*,#], Olubukola O. Babalola[c,d], Reinhard Böhmer[e], Don A. Cowan[f], Kgabo L.M. Moganedi[g], Itumeleng Moroenyane[h], Jerolen Naidoo[i,j,k], Abigail Nieves Delgado[l], Joram M. Posma[m], Leopoldo N. Segal[n] & Mathabatha E. Setati[a,o]

[a.] African Microbiome Institute, Division of Molecular Biology and Human Genetics, Department of Biomedical Sciences, Faculty of Medicine and Health Sciences, Stellenbosch University, Cape Town 7500, South Africa

[b.] DSI-NRF Centre of Excellence for Biomedical Tuberculosis Research; South African Medical Research Council Centre for Tuberculosis Research; Division of Molecular Biology and Human Genetics, Faculty of Medicine and Health Sciences, Stellenbosch University, Cape Town 7500, South Africa

[c.] Food Security and Safety Focus Area, Faculty of Natural and Agricultural Sciences, North-West University, Private Bag X2046, Mmabatho 2735, South Africa

[d.] Department of Life Sciences, Imperial College London, Silwood Park Campus, Buckhurst Road, Ascot, Berkshire SL5 7PY, UK

[e.] PathCare laboratories, Goodwood, Cape Town, South Africa.

[f.] Centre for Microbial Ecology and Genomics, Department of Biochemistry, Genetics and Microbiology, University of Pretoria, Pretoria 0002, South Africa

[g.] Department of Biochemistry, Microbiology and Biotechnology, University of Limpopo, Sovenga 0727, South Africa

[h.] The Plant Holobiont Lab, Department of Botany and Zoology, Faculty of Science, Stellenbosch University, Stellenbosch 7602, South Africa

[i.] Council for Scientific and Industrial Research, Future Production Chemicals Cluster, Pretoria 0002, South Africa

[j.] Department of Biochemistry, Genetics and Microbiology, University of Pretoria, Pretoria 0002, South Africa

[k.] Department of Human Biology, Faculty of Health Sciences, University of Cape Town, Cape Town 7925, South Africa

[l.] Freudenthal Institute, Utrecht University, The Netherlands.

[m.] Section of Bioinformatics, Division of Systems Medicine, Department of Metabolism, Digestion and Reproduction, Hammersmith Hospital campus, Imperial College London, London, UK.

[n.] Division of Pulmonary, Critical Care, and Sleep Medicine, NYU Grossman School of Medicine, New York, USA.

[o.] South African Grape and Wine Research Institute, Stellenbosch University, Stellenbosch 7602, South Africa

*Contributed equally (joint first co-authors); name order chosen alphabetically

#Corresponding author

Table S1. The program of the 3rd African Microbiome Symposium Workshop, Cape Town, South Africa

**Exploring cutting-edge tools and technologies for microbiome research (Host: Separations)**	
**TOPIC**	**SPEAKERS**
Welcome and Separations introduction	Simoné van Zyl
Developing a gut model using 3D bioprinting	Tash Vogt & Talita Turvey
Innovative environmental and clinical sampling and nucleic acid isolation technologies	Sibylle von Boetticher & Simoné van Zyl
Full-length short-read 16S rRNA amplicon sequencing and assembly using the Illumina iSeq100	Lauren Martin (Stellenbosch University)
Illumina metagenomic sequencing technologies	Natasha Kitchin
Illumina BaseSpace Sequence Hub Bioinformatic tools for metagenomic analysis	Natasha Kitchin
Concluding remarks & Closing	Gareth Hughes & Keith Ryan

Table S2. The academic program of the 3rd African Microbiome Symposium, Cape Town, South Africa

**DAY 1**	
**WELCOME & PLENARY SESSION**	
Welcome and introduction by Stellenbosch University Deputy Vice Chancellor	Wim de Villiers, SU
Keynote | Unraveling host-microbe interactions in the lower airways though multi-omics	Leopoldo Segal, NYU
**SESSION 1: Cutting-edge insights into microbiomes and human health Chair: Sian Hemmings**	
The CSIR Microbiome Mapping Initiative	Jerolen Naidoo, CSIR
Intestinal microbiome changes in response to amino acid and micronutrient supplementation: Secondary analysis of the AMAZE Trial	Monica Mweetwa, TROPGAN
Exploring the use of nanopore metagenomic sequencing for the detection and characterisation of infectious causes of disease	Malefu Moleleki, Stellenbosch University
**SESSION 2: Microbiomes in agrifood systems Chair: Florian Bauer**	
Rhizospheres in the Global South	Olubukola Oluranti Babalola , North-West University
Harnessing African indigenous knowledge and microbiota in the production of marula fruit wine and accessorial products	Kgabo Moganedi, University of Limpopo
Unlocking the Power of the Extended Phenotype: Pioneering Soybean Resilience Through Microbiome-Driven Drought Defence	Itumeleng Moroenyane, Stellenbosch University
Fermentative capacity development in ostrich chicks: implications for nutrition and gut health	Annelise Botes, Stellenbosch University

Indole acetic acid production and nutrient solubilisation as indicators of plant growth-promotion potential of two biocontrol microbial consortia	Amber Africa, Stellenbosch University
Platinum sponsor presentation 1 | Inqaba biotec	
**SESSION 3: Flash talks**<br>**Chair: Dr Joram Posma**	
Whole genome sequence analysis of multi-drug resistant and biofilm-forming *Staphylococcus haemolyticus* isolated from bovine milk	Daniel Ajose, North-West University
Investigating an agnostic metagenomic virus discovery tool and evaluating the performance of Ion Torrent versus Oxford Nanopore Technology sequencing	Tshegofatso Mahlangu, Stellenbosch University
Establishment of World Microbiome Partnership – joining efforts towards One Microbiome Health	Indré Karciauskaite, World Microbiome Partnership
Molecular identification and the effect of abiotic stress on bioactive metabolite production for endophytic *Bacillus* isolates from *Solanum nigrum*	Matsobane Tlou, North-West University
The vaginal microbiome of black African women with dysbiosis and HIV infection in late pregnancy	Mathys Redelinghuys, University Of The Witwatersrand
Antibacterial and Antidiabetic Potential of *A. betulina* Extracts in Diabetic Foot Ulcers: *In Vitro* and *In Silico* Studies	Zanele Mathenjwa, University Of Zululand
Marine bacterial symbionts' immunomodulatory and antimycobacterial activity	Funanani Thagulisi, Stellenbosch University
Association between systemic immune marker profiles and microbiome diversity during TB disease	Loide Shipingana, Stellenbosch University
A Meta-Analysis of Gut Microbiome Research in Malnourished African Populations: A Natural Language Processing Approach	Monica Mweetwa, TROPGAN
**Panel discussion | From mapping to function**<br>**Chair: Evodia Setati**<br>L Segal, J Naidoo, K Moganedi, O Babalola, I Moroenyane	
**DAY 2**	
**SESSION 4: Socio-ethical implications of microbiome research**<br>**Chair: Dr Emmanuel Obasa**	
Microbiome research and race in the 'Local South'	Abigail Nieves Delgado, Utrecht University
The ethics of microbiome research	Reinhard Böhmer, UCT/PathCare
**SESSION 5: Advances in microbiome analysis**<br>**Chair: Kristien Nel Van Zyl**	
Literature review and meta-analysis for microbiome research in the language model era	Joram Posma, Imperial College London
The use of bacteriophages as a biocontrol agent against multi drug-resistant *Escherichia coli* O157:H7: Molecular Characterization and Safety Properties.	Bukola Oluwarinde, North-West University
Metagenomic surveillance to predict changes in pathogen profiles on the neonatal platform at Tygerberg Hospital	Kafilat Taiwo Salvador-Oke, Stellenbosch University
Characterization of pomegranate-associated fungal microbiota and identification of fermentative yeasts across different locations	Kasiemobi Ezeora, Stellenbosch University

**SESSION 6: Microbiomes in terrestrial and aquatic ecosystems**<br>**Chair: Itumeleng Moroenyane**	
Functional Microbiomics of Namib Desert Soils	Don Cowan, University of Pretoria
Biogeographical patterns in seagrass sedimentary and rhizosphere microbiomes	Andrew Ndhlovu, Stellenbosch University
Strategic identification and tracking of beneficial microorganisms for the construction of synthetic microbial communities	Tacha-Marie Joubert, Stellenbosch University
Exploring the stool metabolome in symptomatic people with or without tuberculosis	Luis Mur, Aberystywyth University
Platinum sponsor presentation 2 | Qiagen	
**Panel discussion | African footprint in microbiome research**<br>**Chair: Charissa Naidoo**<br>D Cowan, A Delgado, R Böhmer, J Posma	
**Prize giving: Best student oral and poster presentations**	
Best oral presentation	Andrew Ndhlovu
Best poster presentation	Tebogo Masetlana
**Closing**	
Closing address	Thulani Makhalanyane, Stellenbosch University
**E-POSTER PRESENTATIONS**<br>Posters broadcasted throughout the symposium	
Unravelling the pathotype, virulome, and resistome of bacteria isolated from faeces	Tesleen Abolarinwa, North-West University
Whole genome sequence analysis of multi-drug resistant and biofilm-forming *Staphylococcus haemolyticus* isolated from bovine milk	Daniel Ajose, North-West University
Investigating the antibacterial activity of novel silver phosphine complexes against bacteria that contribute towards biofilm formation in wounds	Aarifah Battey, University of the Witwatersrand
Bacterial topology of the respiratory tract, including site-of-disease in people with pulmonary tuberculosis	Tinaye Chiyaka, Stellenbosch University
Exploring health promoting components in traditionally fermented foods and its impact on the gut microbiome for positive health benefits amongst the Aawambo ethnic group in Namibia	Steven Denk, University of Namibia
Gut microbiome profiles associated with posttraumatic stress, depressive and anxiety symptoms in South Africans - the SANeuroGut study	Robert Glennon, Stellenbosch University
Evaluating metagenomic surveillance of antimicrobial resistance in a neonatal hospital environment	Khensani Hattingh, Stellenbosch University
Establishment of World Microbiome Partnership – joining efforts towards One Microbiome Health	Indré Karciauskaite, World Microbiome Partnership
Sulfamethoxazole-Trimethoprim: impact on plant microbiome and resistomes	Caryn Kgokonyane Lenonyane, North-West University

Investigating an agnostic metagenomic virus discovery tool and evaluating the performance of Ion Torrent versus Oxford Nanopore Technology sequencing	Tshegofatso Mahlangu, Stellenbosch University
Short-read full-length 16S rRNA amplicon sequencing for characterisation of the respiratory bacteriome of captive and free-ranging African elephants	Lauren Martin, Stellenbosch University
Comparative holobiont dynamics in drought resilience: exploring the role of Fynbos microbiota in *Sutherlandia frutescens* and *Glycine max*	Tebogo Masetlana, Stellenbosch University
Antibacterial and antidiabetic potential of *A. betulina* extracts in diabetic foot ulcers: in vitro and in silico studies	Zanele Mathenjwa, University of Zululand
Metagenomic and metabolomic characterisation of faecal samples of wild badgers with and without bovine tuberculosis	Luis Mur, Aberystywyth University
Integrative analysis of signalling peptides, rhizosphere microbes, and epigenetic modifications to enhance soybean tolerance to drought and salinity stress	Pirtunia Nyadzani Mushadu, Stellenbosch University
A meta-analysis of gut microbiome research in malnourished African populations: a natural language processing approach	Monica Mweetwa, TROPGAN
Association between systemic immune marker profiles and microbiome diversity during TB disease	Loide Shipingana, Stellenbosch University
Marine bacterial symbionts' immunomodulatory and antimycobacterial activity	Funanani Thagulisi, Stellenbosch University
Molecular identification and the effect of abiotic stress on bioactive metabolite production for endophytic *Bacillus* isolates from *Solanum nigrum*	Matsobane Tlou, North-West University
